# Acute Motor Axonal Neuropathy in a Child With Atypical Presentation: A Case Report

**DOI:** 10.1097/MD.0000000000000392

**Published:** 2015-01-26

**Authors:** Kyung Soo Lee, Seung Hoon Han

**Affiliations:** From the Department of Rehabilitation Medicine, Hanyang University College of Medicine, Seoul, Korea.

## Abstract

Acute motor axonal neuropathy (AMAN) is a variant of Guillain–Barre syndrome. It has been reported to have no sensory symptoms and is diagnosed by typical electrophysiological findings of low-amplitude or unobtainable compound muscle action potentials with normal sensory nerve action potentials. However, the authors experienced atypical case of general electrophysiological findings of AMAN with pain and paresthesia and presented it. This case implies that clinician should be on the alert to atypical sensory symptoms from the classical presentation of AMAN even if the patient is diagnosed with AMAN electrophysiologically and should consider proper treatment options based on clinical presentations.

## INTRODUCTION

Guillain–Barre syndrome (GBS) is one of the most common causes of acute flaccid paralysis in infants and children.^1^ Classic symptoms and presentation of GBS are ascending motor weakness peaking within 4 weeks, minimal objective sensory loss, and decreased or absent muscle stretch reflexes. GBS is considered to be an acute immune-mediated polyneuropathy with several variations: a classic demyelinating form, acute inflammatory demyelization polyneuropathy (AIDP), acute motor–sensory axonal neuropathy (AMSAN), acute motor axonal neuropathy (AMAN), and Miller–Fisher syndrome.^2^

Among several variations, AMAN is characterized clinically by nearly pure motor syndrome without sensory involvement and final diagnosis of AMAN is based on electrophysiological findings such as decreased amplitude of compound muscle action potential (CMAP) without any evidence of demyelination or change in sensory nerve action potential (SNAP). AMAN is reported more common in China than western countries and the majority of northern Chinese patients with GBS were classified as having AMAN.^3^ In addition, it has not been reported on atypical symptoms except flaccid paralysis of electrophysiologically classified AMAN.

To the authors’ knowledge, this is the first case report of AMAN confirmed by electrophysiological studies that was accompanied by severe pain of the entire body and the authors present the case with a review of relevant literature.

## CASE REPORT

A 3-year-old male with a history of medical treatment for cough, sputum, and rhinorrhea complained of bilateral leg weakness and severe lower back and leg pain for 1 day. He was born at 40 weeks’ gestational age by normal spontaneous vaginal delivery without any perinatal problems. He showed good growth and development and did not suffer from any particular medical illness. Routine immunization had already been administered and any vaccine-associated complications had not been developed.

On admission, the patient complained of severe pain and burning sensation on back and lower extremity as well as flaccid paralysis of the whole body that was worse in the lower extremities. On physical examination, function of all cranial nerves was intact and muscle strength of bilateral proximal upper extremities was grade 1 according to the Medical Research Council (MRC) criteria with no discernible movements elsewhere. Deep tendon reflexes, including patellar and Achilles tendon reflexes, were absent bilaterally. Upper motor neuron signs such as Hoffmann and Babinski signs were absent. There were no signs of ataxia or nystagmus or meningeal irritation signs and no bladder or bowel dysfunction was found at admission.

**FIGURE 1:**
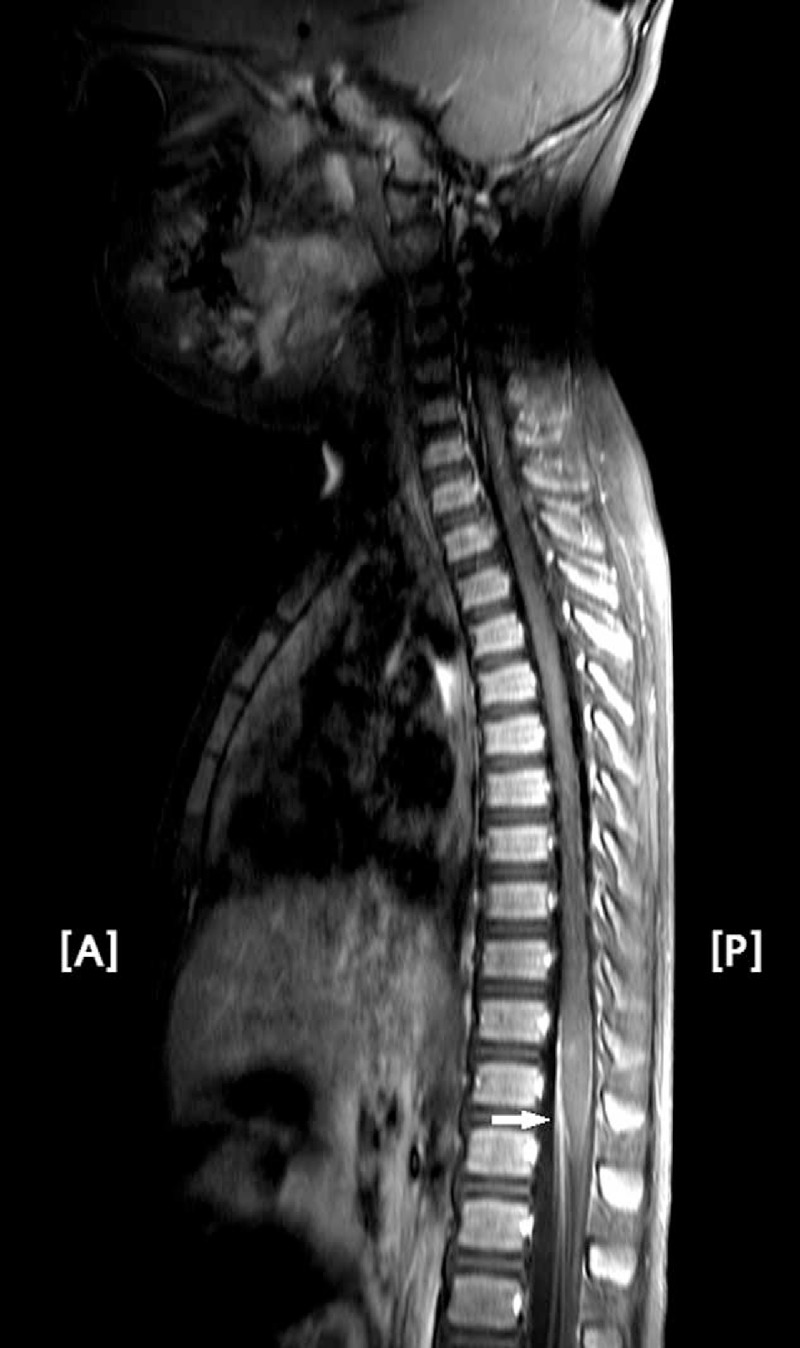
High signal intensity (white arrow) was observed on the cervical and lower thoracic spinal cord as well as the cauda equina in T-1-weighted MRI images after gadolinium injection. A = anterior, MRI = magnetic resonance imaging, P = posterior.

Laboratory studies showed a total white blood cell count of 7500 cells/mm^3^ and C-reactive protein level of 0.17 mg/dL; results of other biochemical blood tests were within normal ranges. Cerebrospinal fluid analysis and magnetic resonance imaging (MRI) of the whole spine were performed for differential diagnosis of acute flaccid paralysis. Cerebrospinal fluid analysis parameters were within normal ranges; however, whole spine MRI after gadolinium injection showed high signal intensity on the cervical and lower thoracic spinal cord as well as cauda equina in the T-1-weighted image (Figure 1). Although albuminocytologic dissociation in cerebrospinal fluid was typical finding in GBS, elevations of protein usually occurred in the second or third week of illness in AMAN, and patients with AMAN may have normal cerebrospinal fluid protein in first week.^4^ In addition, MRI findings were compatible with diagnosis of GBS.

On the basis of above findings, the patient was diagnosed with GBS and intravenous immunoglobulin was administered immediately. Carbamazepine was also administered for pain control. Although the patient did not have any signs of respiratory failure, he was constantly monitored with pulse oximetry due to possibility of progression of respiratory failure. He was referred for electrophysiological studies on his 13^th^ hospital day. SNAP results were within normal limits. Motor nerve conduction studies were not evoked in any of the four limbs (Table 1). F-wave and H-reflexes were not evoked in bilateral tibial nerves. He simultaneously started rehabilitation treatment that included electrical stimulation therapy, range of motion, and strengthening exercises for all limbs.

**TABLE 1:**
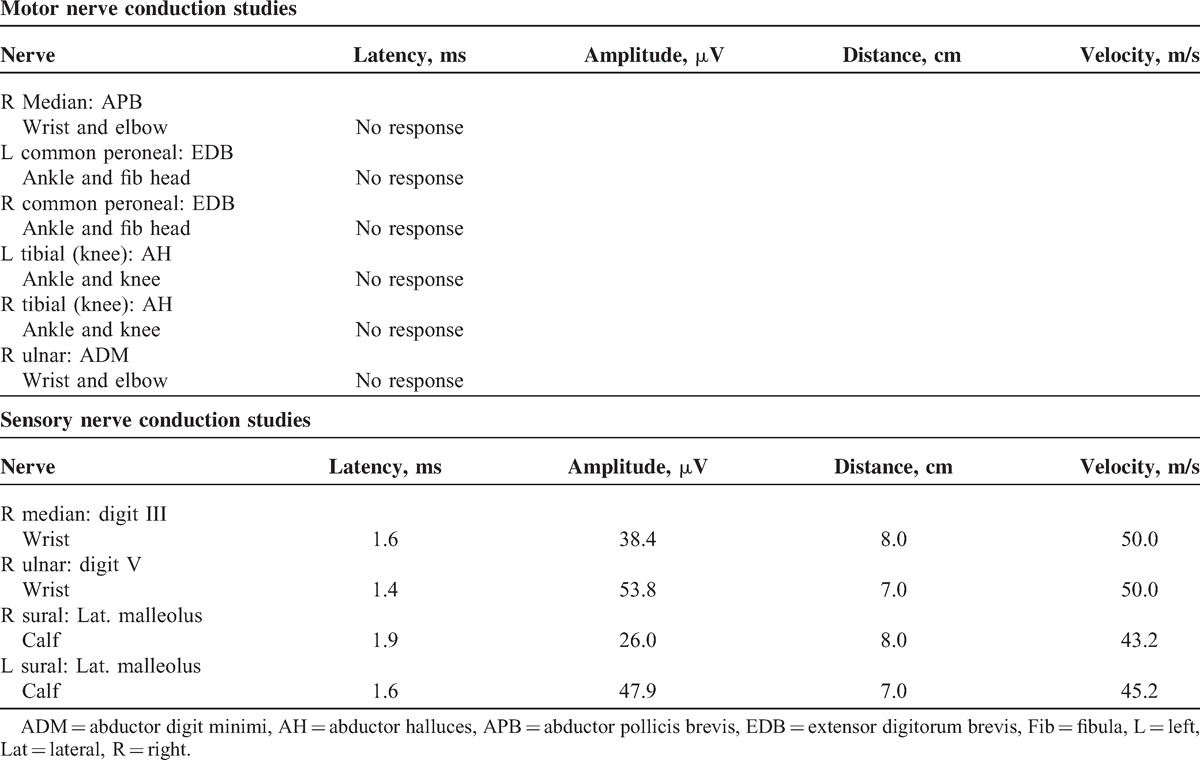
Results of Nerve Conduction Studies on 13th Days after Admission

Pain and paresthesia started to disappear immediately after medication and muscle strength of upper limbs also improved gradually after rehabilitation. At 5 weeks after admission, he could sit on the bed independently for several seconds. Over a period of 2 months, muscle strength of all limbs except bilateral ankle and toe improved gradually to MRC grade 3 and he could pull himself up to stand with assistance of walker. However, electrophysiological findings had not improved compared with previous study. At 4 months after admission, he could walk independently with the application of ankle foot orthosis; however, he could not go up and down the stairs.

Muscle strength of the upper and lower limbs improved gradually over a period of 8 months from the onset of symptoms, and it was assessed as MRC grade 5 and 4, respectively. Fourteen months after onset, the patient showed complete recovery of muscle strength of all 4 limbs except for mild weakness of all toes. Electrophysiological findings had also improved relative to the previous studies. CMAP of the right peroneal nerve was evoked on recording at the tibialis anterior muscle, and nerve conduction velocities were also higher, albeit still lower than normal values.

## DISCUSSION

GBS can be classified into 4 subgroups based on clinical and electrophysiological criteria: AIDP, AMAN, AMSAN, and Miller–Fisher syndrome.^2^ AIDP is characterized by generally demyelinating features, and the amplitudes of distal CMAPs are at least 10% higher than the lower limit of normal.^2^ In contrast, in AMAN and AMSAN, the amplitudes of distal CMAPs are 10% less than the lower limit of normal. If these electrophysiological deficits are limited to purely motor fibers, AMAN is diagnosed, whereas if additionally sensory fibers are affected, AMSAN is diagnosed. Miller and Fisher described a variant of GBS characterized by the triad of acute ophthalmoplegia, ataxia, and areflexia; this variant is now known as Miller–Fisher syndrome.^2^

When the patient was initially referred to rehabilitation department, the authors assumed a diagnosis of AIDP or AMSAN because he complained of severe back and bilateral leg pain as well as flaccid paralysis of all limbs. However, electrophysiological studies showed typical findings of AMAN; CMAPs were not evoked in any of the 4 limbs, whereas SNAPs showed a normal shape and amplitude. In a previous report, AMAN was characterized by rapidly progressive ascending quadriparesis without any sensory involvement^4^; there have been no prior reports that described AMAN with sensory symptoms.

The authors suggest 2 possible pathophysiologic mechanisms of patient's sensory symptoms. First, his sensory symptoms may have been caused by minute injuries to small sensory nerve fibers that were too small to be detected by routine electrophysiological tests. In this regard, Stewart *et al*. defined small-fiber neuropathy as a peripheral neuropathy manifesting as paresthesias with findings of small-fiber dysfunction on neurologic examination. They also reported that routine electrophysiological tests of small-fiber neuropathy were generally normal because they represented the function of large myelinated nerve fibers.^5^ However, in this case, although all SNAPs were evoked with normal form, his diagnosis with AMAN rather than small-fiber neuropathy was reasonable because CMAPs were not evoked in any of the 4 limbs.

Second, immunologic reaction of the whole spinal cord may have caused temporal polyradiculopathy or myelopathy and may have induced severe pain throughout the body, especially the lower back and bilateral legs. Whole spine MRI revealed high signal intensity on the cervical and lower thoracic spinal cord as well as cauda equina after gadolinium injection, indicating inflammatory changes inside the thecal sac. Iwata and Utsumi suggested the mechanism of enhancement might be breakdown of the blood-nerve barrier inside the thecal sac.^6^ For differential diagnosis of diseases such as polyradiculopathy or myelopathy, needle electromyography and evoked potential testing should have been done as initial electrophysiological tests. However, these tests could not be performed due to refusal by the parents.

Tripathi *et al.* recommended carbamazepine as an adjuvant to treat pain in GBS patients during the recovery phase in the intensive care unit to reduce narcotic requirements.^7^ Carbamazepine was prescribed to relieve severe pain and paresthesia immediately, which slowly subsided and disappeared completely after 2 months. Because this case complained of severe pain and paresthesia at early stage of disease, the authors could manage those symptoms by prescribing proper medications.

Muscle strength of whole body also had recovered to MRC grade 5; however, CMAPs of all 4 extremities were still abnormal at 14 months after symptom onset. Electrophysiological findings of AMAN are not always markers of poor prognosis, and almost all the patients with severe AMAN who recover slowly over the first 6 months are eventually able to walk independently, although some require several years.^8^ Furthermore, in children, recovery tends to be more rapid and more likely to be complete and death is exceptional.^9^ In this patient, it took 4 months for him to walk with ankle foot orthosis and 8 months for the motor paralysis to resolve.

## CONCLUSIONS

This case implies that clinician should be on the alert to atypical sensory symptoms from the classical presentation of AMAN even if the patient is diagnosed with AMAN electrophysiologically and should consider proper treatment options based on clinical presentations.
